# The effect of curcumin on embryonic in vitro development in experimental polycystic ovary syndrome: An experimental study

**DOI:** 10.18502/ijrm.v19i11.9915

**Published:** 2021-12-13

**Authors:** Yousef Nasiri Bari, Vahab Babapour, Abbas Ahmadi, Morteza Zendehdel Kheybari, Ghasem Akbari

**Affiliations:** ^1^Department of Basic Sciences, Faculty of Veterinary Medicine, Science and Research Branch, Islamic Azad University, Tehran, Iran.; ^2^Department of Basic Sciences, Faculty of Veterinary Medicine, Tehran University, Tehran, Iran.; ^3^Department of Basic Sciences, Faculty of Veterinary Medicine, Urmia University, Urmia, Iran.; ^4^Department of Clinical Sciences, Faculty of Veterinary Medicine, Science and Research Branch, Islamic Azad University, Tehran, Iran.

**Keywords:** Polycystic ovary syndrome, In vitro fertilization, Curcumin, Mice.

## Abstract

**Background:**

Polycystic ovary syndrome (PCOS) is a common disease in women. Some plant compounds which have antioxidant properties, such as curcumin, may be useful for these patients when delivered orally or in vitro.

**Objective:**

The aim of this study was to evaluate the impact of PCOS on oocyte quality and the effect of curcumin on in vitro fertilization of oocytes.

**Materials and Methods:**

In this experimental study, Naval Medical Research Institute mice aged six to eight wk were used. Mice were divided into five experimental groups (control, experimental PCOS, curcumin 6, 12 and 24 μM). To induce experimental PCOS, estradiol valerate (100 mg/kg, IP) was injected. The total antioxidant capacity and production of malondialdehyde in ovarian tissue and blood serum were evaluated in all groups. Finally, 6, 12 and 24 μM of curcumin were added to the culture medium of the PCOS group oocytes and development in the different groups was evaluated.

**Results:**

A high percentage of oocytes for fertilization were not in good condition in terms of number and quality in the group of PCOS. The addition of curcumin to the embryo culture medium was associated with a higher percentage of fertilized oocytes, two-cells and blastocysts. This increase was significant at a concentration of 24 μM (p ≤ 0.01).

**Conclusion:**

Given that adding curcumin seemed to improve fetal growth and prevent the harmful effects of oxygen free radicals on the culture medium, it is recommended to add a certain concentration of curcumin under normal conditions without oxidative stress.

## 1. Introduction

Polycystic ovary syndrome (PCOS) is a complex condition, due to the associated high levels of androgens (testosterone, androstenedione and dehydroepiandrostenedione), irregularities in endometrial periods and the presence of small cysts on one or both ovaries (1, 2). This disease is called a pathology of the 20 ${}^{\mathrm{th}}$ century (3). PCOS can have many negative effects on the body, such as hyperinsulinemia, insulin resistance, obesity, and hypertension, and in the long run, can lead to endometrial hyperplasia and cardiovascular disease (4).

In these women, the concentration of insulin and insulin-like factors may be higher, which increases the synthesis of androgens in the single cells and thereby enhances luteinizing hormone function (5). Too much androgen leads to the breakdown of meiosis and mitosis and ultimately to the incorrect maturation of oocytes (6).

High levels of androgens negatively affect the quality of the fetus, and its quality gradually declines with age. The fetuses of patients with PCOS who are over the age of 35 are usually at risk of developing arrest (7). The embryos obtained from these patients are delayed in the formation of two pronuclei and the four to eight stages of cell proliferation, but the effect of this delay on embryo growth, implantation, and pregnancy rate is not significant (8). In contrast, the prevalence of miscarriage is higher in these women (9). Production of reactive oxygen species (ROS) seems to be one of the main reasons for stalled growth of embryos cultured in the laboratory. ROS can stop meiosis in the oocyte, inhibit embryo growth and lead to cell death (10, 11).

The impact of oxidative stress (OS) on fertility is a major research topic in many countries. Thus, the extent of injury or its advantage to the living organism is directly related to the site of oocyte fertilization (12). OS is the result of an imbalance between ROS and antioxidants (13). Curcumin has been evaluated for its many properties, and its efficacy in the treatment of PCOS has been examined in animal models and clinical studies (14). Curcumin is a polyphenolic compound derived from the root of the plant *Curcuma longa* (15, 16). *Curcuma longa* has a wide range of biological and pharmacological uses (17).

Consumption of foods containing curcumin also has protective effects on most organs of the body (18). Curcumin is used to prevent various diseases including cancer of the colon, breast and pancreas (19). There are not many reports of curcumin toxicity for oocyte maturation and fertility and embryonic cell development (20). Therefore, the aim of this study was to compare the effect of non-oral curcumin at different concentrations in a culture medium on the success rates of in vitro fertilization (IVF) oocytes from experimental polycystic ovaries induced by estradiol valerate (EV), compared with control samples.

## 2. Materials and Methods 

For this experimental study, 60 Naval Medical Research Institute (NMRI) female mice aged six to eight wk with an average weight of 20 gr were obtained from the Laboratory Animal Research and Maintenance Center of Urmia University of Medical Sciences. This study was conducted from April to December 2019 in accordance with the ethical principles of working with laboratory animals in the IVF laboratory of the Faculty of Veterinary Medicine, Urmia University, Urmia, Iran. The mice were kept during polycystic development under standard conditions with a temperature of 22 ± 2°C, 30-60% humidity, an optical cycle of 14 hr of light and 10 hr of darkness, and hygienic conditions with water and food available freely. The animals were randomly assigned to either the control group or the experimental PCOS group. The experimental PCOS group received a single intraperitoneal injection of EV for polycystic ovarian induction (100 mg/kg per BW, Aboreyhan, Iran). The mice developed polycystic ovaries 60 days after EV injection and PCOS induction was confirmed by ovarian tissue examination. Oocytes from the mice were divided into five experimental groups, all of which were cultured in a human tubal fluid (HTF, Sigma, United States) medium containing 4 mg/ml bovine serum albumin (BSA, Sigma, United States) for fertilization and embryo culture: 1) control group oocytes; 2) experimental PCOS group oocytes; 3) experimental PCOS group oocytes with the addition of 6 µM curcumin; 4) experimental PCOS group oocytes with the addition of 12 µM curcumin; and 5) experimental PCOS group oocytes with the addition of 24 µM curcumin.

To confirm the induction of PCOS, the physical condition of the animal was examined. Mice with PCOS were severely underweight and cystic on the ovaries under the loop. A histological study was also performed to further confirm the PCOS.

To determine the level of lipid peroxidation, the amount of malondialdehyde (MDA) production in the ovarian tissues and serum was measured using a thiobarbituric acid (TBA) reaction. For this purpose, each ovarian specimen was homogenized in a 150 ml potassium chloride solution and then the resulting mixture was centrifuged at 3000 rpm for 10 min. 500 µl of homogenous supernatant for each ovarian sample and 200 µl of serum for each serum sample were mixed with 3 ml of phosphoric acid and then vortexed, and 1 ml of TBA was added to the samples. The samples were incubated for 45 min at 100°C and then cooled in ice. Subsequently, the samples were centrifuged at 3000 rpm for 10 min after adding 3 ml of N-butanol. Optical absorption of the supernatant at 532 nm was measured by spectrophotometer and calculated based on the standard calibration curve of MDA in nM per mg protein.

To evaluate the total antioxidant capacity (TAC) in the ovarian tissues and blood serum, the ferric reducing ability of plasma method was used. In this method, in acidic pH created by a buffer, an aqueous color was produced by the reduction of (Fe ${}^{++}$ ) ions of TPTZ-Fe3 ${}^{+}$ complex and their conversion to (Fe2 ${}^{+}$ ) ions, which was measured spectrophotometrically at 493 nm. After inducing PCOS, a hormone injection was given in two stages to stimulate ovulation and obtain a mature oocyte.

In the first step, the mice were injected intraperitoneally with 0.1 ml 7.5 IU of pregnant mare serum gonadotropin (PMSG), (Folligon, the Netherlands) at around 7:00 PM. 46-48 hr after the first injection, the mice were injected intraperitoneally with 0.1 ml 7.5 IU of human chorionic gonadotropin (hCG), (Folligon, the Netherlands). Ovulation usually occurred about 10 to 13 hr after the hCG injection.

A T6 medium supplemented with 4 mg of BSA was used to maintain the spermatozoa that were harvested to evaluate the desired parameters, and another culture medium with 1 ml of HTF supplemented with 4 mg of BSA was used to fertilize the sperm and oocytes. One day before fertilization, drops of culture medium containing different concentrations of curcumin (6, 12 and 24 µM) were prepared, and these were placed 12 hr before fertilization for equilibrium in a 5% CO ${}_{2}$ incubator at 37°C.

Sperm were selected from 8-12 wk old male mice with an average weight of 25-30 gr, facilitated by anesthesia injection. Sperm collection began one hr before oocyte collection from female mice, and after opening the abdomen, the epididymal tail and part of the deferens were separated under sterile conditions and placed in a 6 cm petri dish containing pre-prepared HTF medium with 4 mg of BSA. Then, by dissecting, the sperm were removed from the cauda epididymis and placed in a CO ${}_{2}$ incubator for 30 min to allow the sperm to undergo capacitation in the culture medium. After half an hr, the sperm were washed and the swim up method was used to extract the removable sperm which were placed in an incubator for one hr at 37°C. All samples were centrifuged at 1500 rpm for 15 min. After centrifugation and collection of sperm at the bottom of the tube, the supernatant formed was removed and less than 0.5 ml of culture medium was left at the top of the sediment and placed at 37°C for 30-45 min for swim up. Sperm that had a natural shape and proper movement came to the surface and were placed inside their top culture medium. Then 20 μl of the supernatant was placed on the slide and evaluated with a light microscope, and the sperm parameters were recorded. The Eosin-Nigrosin test was used to count the sperm using a hemocytometer and to evaluate the viability of the sperm; 20 μl of the desired sperm sample was dissolved on a clean slide with 20 μl of Eosin solution and after 20 to 30 sec, 20 μl of Nigrosine dye solution was added. After preparing the smear from the solution and drying the slides, the morphology, viability and mortality were examined using a light microscope with × 40 magnification. Subsequently, the sperms which had over 95% morphological parameters and over 80% motility and viability were used for fertilization.

After the hCG injection, female mice in the IVF laboratory were relaxed after anesthesia and the fallopian tubes were removed and placed in an HTF culture medium containing 4 mg/ml of BSA at 37°C. The oocytes were then separated from the fallopian tubes by dissection and after washing, the oocytes were transferred to fertilization medium droplets containing an HTF culture medium with 4 mg/ml of BSA and 1 million motile sperm per drop of fertilization. 0.5 ml of culture medium was added to this and mineral oil was poured onto the droplets inside the petri dish. Fertilization was performed for five to six hr in an incubator at 37°C with 5% CO ${}_{2}$ and a zygote was obtained. 10 mM of commercial curcumin powder (Merck, Germany) was dissolved in 1 mg/ml of dimethyl sulfoxide (DMSO) and then stock was prepared and stored in the dark at -20°C. This stock was then used to prepare different levels of curcumin to be added to the culture medium. IVF zygotes were immersed in drops of HTF culture containing 4 mg/ml of BSA by adding concentrations of 6, 12 or 24 μM curcumin under mineral oil. All groups were cultured for 120 hr and then the different stages of embryonic development were evaluated using a contrast phases microscope. Embryos in each group were examined for fragmentation and cytoplasmic vesicles. Embryos were classified as one of three types: type I - embryos with lysis and fragmentation; type II - embryos with lysis and fragmentation in a number of blastomeres; and type III - embryos with a small amount of lysis and fragmentation in blastomeres and cytoplasmic vesicles.

### Ethical considerations

This research was approved by the Ethics Committee in Biomedical Research of Islamic Azad University, Science and Research Branch (Code: 8/29/58211).

### Statistical analysis

Statistical analysis of the fertilization rate, percentage of two-cell embryos, percentage of blastocysts, embryo lysis and fragmentation rate data was carried out using Minitab software (Minitab Co., United States). Comparison of the mean MDA and TAC test of the serum and ovarian tissue of the control and PCOS samples were analyzed using the Statistical Package for the Social Sciences software version 16 (SPSS, United States) and *t* test. Data were considered significant at p < 0.01. The means and standard deviations were also obtained.

## 3. Results

**Figure 1:**
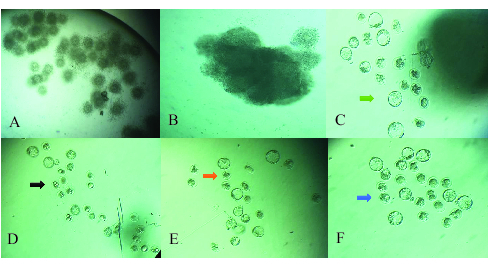
Oocyte embryos in different groups were evaluated after 120 hr of embryo culture with an inverted microscope and magnification of × 100. A: Control group, Cumulus-oocyte complex (COC) with good quality; B: Oocytes in the PCOS group with cumulus masses of poor quality and compressed cumulus masses (Compact); C: Oocytes in PCOS group without cumulus masses and a large number have lysed and fragmented (Green arrow); D: Embryos in the control group where many have reached the blastocyst stage and embryos are good quality in terms of morphology (Black arrow); E: Embryos in the PCOS group where only a few have reached the blastocyst stage and many arrested in various stages of embryonic development, and the resulting embryos are not good quality in terms of morphology (Red arrow); F: Embryos in the PCOS group in which curcumin was added to the embryonic culture and the number of blastocysts obtained was higher in comparison with PCOS, and the resulting embryos are good quality in terms of morphology (Blue arrow).

In this study, the ferric reducing ability of plasma method was used to evaluate the TAC of ovarian tissue and blood serum. TAC, ovarian tissue and blood serum were significantly lower in the PCOS group compared to the control group. To determine lipid peroxidation and MDA production, ovarian tissue and serum levels were measured using the TBA reaction. The amount of MDA, which indicates the level of lipid oxidation, was significantly higher in the ovarian tissue and serum of the PCOS group compared to the control group (Table I). In this study, the number of oocytes in the PCOS group was significantly different compared to the control group; a high percentage of oocytes were of poor quality for IVF; and many oocytes had pressed cumulus-oocyte complexes and masses, and were seen as lysed. A smaller percentage of oocytes were of good enough quality for fertilization compared with in the control group (p ≤ 0.01). In the analysis of the percentage of oocytes for fertilization, a significantly lower percentage was observed in the PCOS group than in the control group, and the highest embryo growth retardation was of type I and II (p ≤ 0.01) (Table II, Figure 1). Adding different concentrations of curcumin to the culture medium was associated with a higher percentage of fertilization and growth quality, and altered morphology.

The percentage of fertilization in the culture medium was higher when curcumin was added: the concentration was 89.64% when 6 µM was added, 91.16% when 12 µM was added, and 91.40% when 24 µM was added, compared to the PCOS group which had 75.35% (p ≤ 0.01). The percentage of two-cell embryos in the culture medium was higher when curcumin was added: the percentage was 85.55%, 83.64% and 86.47% when 6 μM, 12 µM, and 24 µM were added, respectively, compared to the PCOS group which had 59.88% (p ≤ 0.01). The percentage of embryos that had reached the blastocyst stage in the culture medium was also significantly higher when curcumin was added: the concentration was 38.15%, 34.54% and 37.16% when 6 μM, 12 µM, and 24 µM of curcumin were added, respectively, compared to the PCOS group, which had 27.78% (p ≤ 0.01). Adding different concentrations of curcumin was associated with a lower percentage of arrested embryos compared to the PCOS group. Most of the embryos with high lysis and fragmentation were type I and II, and low lysis and fragmentation embryos in the presence of curcumin were type III, which showed the antioxidant effects of curcumin on embryo damage during fertilization and laboratory culture. These differences in lysis, fragmentation, percentages, and arrested embryos in the presence of curcumin were significant compared to the PCOS group (p ≤ 0.01) (Table II).

**Table 1:** Comparison of TAC and MDA results for ovarian and serum samples in the control and PCOS groups


	**TAC (nmol/mg)**		**MDA (nmol/mg)**	
**Group**	**Ovarian samples **	**Serum samples**	**Ovarian samples **	**Serum samples**
**Control**	0.0793 ± 0.0032 ${}^{a*}$	0.1382 ± 0.0491 ${}^{a**}$	20.870 ± 4.320	3.480 ± 0.522
**PCOS**	0.0214 ± 0.0087	0.0710 ± 0.0042	86.750 ± 13.240 ${}^{a**}$	5.279 ± 0.690 ${}^{a**}$
Data presented as Mean ± SD. TAC: Total antioxidant capacity, MDA: Malondialdehyde, PCOS: Polycystic ovary syndrome. *P ≤ 0.01, **P < 0.01, ${}^{a}$ A significant difference between groups				

**Table 2:** Comparison of the effect of adding different doses of curcumin to the in vitro culture medium of experimental PCOS mice for in vitro fertilization and embryo development


**Group**	**Suitable oocytes N**	**Fertilization N (%)**	**Two-cell N (%)**	**Blastocysts N (%)**	**Arrested embryos N (%)**	**Type I N (%)**	**Type II N (%)**	**Type III N (%)**
**Control**	173	165 (95.38)	152 (92.12)	101 (61.21)	64 (38.79)	2 (1.21)	5 (3.03)	57 (34.54)
**PCOS**	215	162 (75.35) ${}^{a*}$	97 (59.88) ${}^{a*}$	45 (27.78) ${}^{a*}$	117 (72.22) ${}^{a*}$	56 (34.57) ${}^{a*}$	39 (24.07) ${}^{a*}$	22 (13.59) ${}^{a*}$
**PCOS + Cur 6 µM**	193	173 (89.64) ${}^{a**b*}$	148 (85.55) ${}^{b*}$	66 (38.15) ${}^{a*b**}$	107 (61.85) ${}^{a*b**}$	15 (8.67) ${}^{ab*}$	22 (12.72) ${}^{ab*}$	58 (33.53) ${}^{b*}$
**PCOS + Cur 12 µM**	181	165 (91.16) ${}^{b*}$	138 (83.64) ${}^{a**b*}$	57 (34.54) ${}^{a*}$	108 (65.46) ${}^{a*}$	10 (6.06) ${}^{a**b*}$	27 (16.36) ${}^{a*}$	71 (43.03) ${}^{b*}$
**PCOS + Cur 24 µM**	186	170 (91.40) ${}^{b*}$	147 (86.47) ${}^{b*}$	63 (37.16) ${}^{a*}$	107 (62.49) ${}^{a*}$	14 (8.23) ${}^{ab*}$	22 (12.94) ${}^{ab*}$	71 (41.76) ${}^{b*}$
Data presented as n (%). PCOS: Polycystic ovary syndrome, Cur: Curcumin. *P < 0.01, **P < 0.01, Different superscript letters indicate significant differences as follows - ${}^{a}$ Compared to control group, ${}^{b}$ Compared to PCOS								

## 4. Discussion 

In the present study, a high percentage of oocytes were of low quality in terms of IVF: the percentages of fertilization, two-cell embryos and blastocysts were lower in the PCOS group compared to the control group. A significantly higher percentage of embryos that were arrested at different stages of development were found in the PCOS group compared to in the control group, which had the highest embryonic growth cessation in type I and II embryos.

These results could indicate destructive effects of PCOS on oocytes, and also that the incubation of gametes in IVF can cause the production of free radicals around the growing zygote and endanger the viability of embryos. We found that adding curcumin to the culture medium as an antioxidant may help to eliminate free radicals and increase the viability of the developing embryos. It is also possible that adding certain concentrations of curcumin to the culture medium will increase sperm quality and improve IVF results.

Therefore, it can be concluded that PCOS and OS have negative effects on IVF results and curcumin, as an antioxidant, can improve these results. The results of this study showed that curcumin can be recognized as an antioxidant. Because women with PCOS often cannot conceive under normal circumstances and therefore benefit from new fertility techniques such as IVF for pregnancy, many changes have occurred in in vitro embryo production. One of the most important causes of failure is the presence of free radicals and ROS (21). On the other hand, the ability of the in vitro embryo to defend itself in different stages of development up to the blastocyst stage appears to vary. Some researchers believe that the embryo's primary antioxidant capacity in the early stages of zygote division depends on the storage of maternal hereditary mRNA in the oocyte, and it is at the embryonic genome activity stage that the fetal gene is able to interact with the oxidants produced. As a result, the embryo's requirements for external antioxidants before and after embryo genome activity appear to vary (22). Oocytes and embryos are protected against oxidative damage by the follicular fluid and fallopian tubes (23).

Therefore, protecting the embryo during in vitro culture against oxygen free radicals by adding antioxidants to the culture medium is essential to increase embryo growth (24). Curcumin is one of the antioxidants that can be used to improve the level of fetal growth and development. It can be administered both orally and in vitro in patients with PCOS. However, few field and laboratory studies have been performed on human and animal models (25).

Adding curcumin as a plant compound in certain concentrations to the embryonic culture medium can improve embryo growth and development. Curcumin can reduce ROS by inhibiting methylglyoxal, but in large amounts can disrupt the biochemical events of early embryonic cells and blastocysts. Some studies have reported that curcumin at higher concentrations in different laboratory stages has toxic effects on embryo development (15, 19, 20, 26, 27).

Along with ROS, high doses of curcumin can have destructive effects on blastocyst cells. It also interferes with mitochondrial signals and can therefore disrupt fetal growth (15, 27). In one study, the beneficial effects of curcumin along with letrozole on polycystic ovaries in Wistar rats were investigated. The results showed that PCOS caused abnormalities in sex steroids and fat, glucose and hemoglobin levels, and decreased antioxidants. Curcumin restored all of the natural parameters and eliminated ovarian cysts with protective effects. Curcumin has also been shown to have beneficial effects on mice with PCOS (16).

Most studies show increased OS and ROS production, which are the main causes of peroxidation of phospholipids, proteins, and nucleic acids in oocytes and sperm cells, thereby reducing fertilization and subsequent embryonic development (28, 29).

In this study, curcumin was associated with lower destructive effects relating to the above parameters, a higher rate of fertilization, higher percentages of two-cells and blastocysts, and a lower number of arrested embryos. Finally, it can be concluded that adding certain concentrations of curcumin to the culture medium can play an effective role in embryo development.

## 5. Conclusion

This study showed that the use of curcumin as an antioxidant in IVF culture can improve embryonic growth. 24 µM of curcumin had the greatest effect on embryonic growth. Therefore, curcumin is recommended for improving embryonic growth and development in IVF.

##  Conflict of Interest

The authors declare they have no conflict of interest.
